# Induction of Partial Protection against Foot and Mouth Disease Virus in Guinea Pigs by Neutralization with the Integrin β6-1 Subunit 

**DOI:** 10.3390/v5041114

**Published:** 2013-04-19

**Authors:** Yan Zhang, Yingjun Sun, Fan Yang, Jianhong Guo, Jijun He, Qiong Wu, Weijun Cao, Lv Lv, Haixue Zheng, Zhidong Zhang

**Affiliations:** State Key Laboratory of Veterinary Etiological Biology, National Foot and Mouth Disease Reference Laboratory, Lanzhou Veterinary Research Institute, Chinese Academy of Agricultural Sciences, Lanzhou 730046, China; E-Mails: sallywo8604@yahoo.com.cn (Y.Z.); sunyingjun414@163.com (Y.S.); fanfan026@163.com (F.Y.); gregjh@163.com (J.G.); hejijun1979@163.com (J.H.); wubinbin02@163.com (Q.W.); springcaowei@163.com (W.C.); lvlv-923@163.com (L.L.)

**Keywords:** foot-and-mouth disease, foot-and-mouth disease virus, integrin

## Abstract

The mechanism by which the foot-and-mouth disease virus (FMDV) initiates infection of cells is thought to involve the attachment of the viral capsid to host integrins on the surface of target cells. However, the role of integrins in FMDV infection still needs to be fully understood, although it has been demonstrated that integrin αvβ6 interferes with FMDV in vitro and results in neutralization of its infectivity. In the present study, we describe the cloning and sequencing of suckling mouse integrin β6 and the subsequent expression of two segments of integrin β6 extracellular domains: β6-1 (which contains the ligand-binding domain) and β6-2. Sequencing of the mouse integrin β6 subunit revealed close homology (~90%) with its human counterpart. When recombinant integrin extracellular domains β6-1 and β6-2 formulated with adjuvant were inoculated into guinea pigs, anti-integrin antibody expression was high before FMDV challenge. Interestingly, guinea pigs (50%) inoculated with integrin β6-1 were protected from FMDV infection; in contrast, none of the animals inoculated with integrin β6-2 were protected. This result indicates that an integrin blockade may be able to interfere with FMDV infection in vivo, which raises the possibility that targeting integrin in vivo may be the basis for a new strategy to control FMDV infection.

## 1. Introduction

Foot-and-mouth disease (FMD) is one of the most economically devastating diseases that affects a large number of cloven-hoofed animals, including cattle, pigs, sheep, goats, and camels [1,2]. The etiological agent, FMD virus (FMDV), is the prototype member of the genus *Aphthovirus* within the family *Picornaviridae*, and has seven antigenically distinct serotypes (A, O, C, Asia1, and South African Territories 1, 2, and 3). FMDV is a small, non-enveloped, icosahedral virus, which contains a single-stranded positive-sense RNA genome [3]. The viral capsid is made up of 60 copies each of four structural proteins (VP1 to VP4).

The mechanism by which FMDV initiates infection of cells is thought to involve the attachment of the viral capsid to host integrins on the surface of target cells [4,5]. Interaction of FMDV with integrins requires a highly conserved Arg-Gly-Asp (RGD) triplet (positions 141–143) in the G-H loop of viral capsid protein VP1 [6,7,8,9]. Synthetic peptides spanning residues 133–156 of VP1 faithfully mimic the entire virus particle with regard to interaction with antibodies and also recognition of an integrin receptor [10,11]. Integrins are non-covalently linked heterodimers containing an α and β subunit. Both subunits are type I transmembrane proteins, containing a large extracellular domain, a single transmembrane domain and a small, C-terminal cytoplasmic domain [12]. The ligand-binding site of integrin is formed by non-covalent association of the head (or ligand-binding) domains of the α and β chains. 

Four integrins (αvβ1, αvβ3, αvβ6, and αvβ8) have been identified in vitro as FMDV receptors [7,13,14,15,16,17]. αvβ6 is of particular interest, because it shows high affinity for viruses [18]. Moreover, αvβ6 is expressed constitutively on epithelial cells in areas where lesions form, but not in epithelial cells in non-lesion areas, based on which it is considered to be the major in vivo receptor for FMDV [19]. On investigating the mechanism for αvβ6-mediated infection, it was found that loss of the β6 cytoplasmic domain of the integrin has little effect on the binding of virus to cells [20]. However, the role of αvβ6 in FMDV infection is not fully understood. A previous study demonstrated that soluble αvβ6 was able to interfere with FMDV infection in vitro, resulting in neutralization of viral infectivity [18]. The mechanism is likely to involve inhibition of the binding of the virus to cells *via* binding of integrin to the receptor-binding site on the virus. This result indicates that interference with αvβ6 can neutralize virus infection in vivo. Inhibition of the binding of virus to cells *via* RGD-binding integrins has been invaluable for the study of viral infections, but there have been no studies on the impact of αvβ6-mediated neutralization of FMDV infection in vivo. 

In the present study, we describe the cloning and sequencing of mouse integrin β6 and the subsequent expression of two separate segments of the integrin β6 extracellular domain: (1) the RGD-binding site (β6-1­) and (2) the remaining part (β6-2). We found that the β6-1 segment provided guinea pigs with partial protection against the FMDV challenge. This is a promising result based on which new strategies to control FMDV infection in vivo can be established.

## 2. Results and Discussion

### 2.1. Cloning, Sequencing and Characterization of Suckling Mouse Integrin β6 Subunit

**Figure 1:**
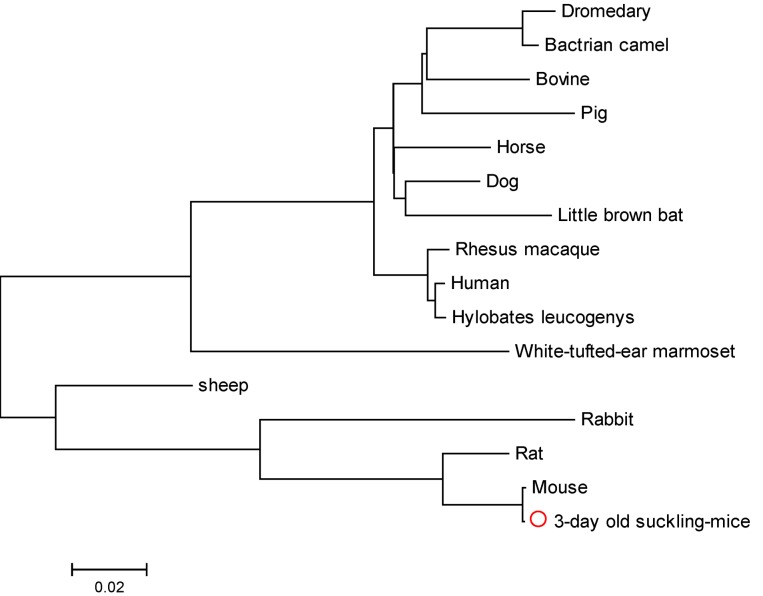
Phylogenetic tree of suckling mouse integrin β6 subunit. The phylogenetic tree was constructed based on the nucleotide sequences, using the neighbor-joining algorithm of MEGA version 5.05. The reference sequences included in the analysis were taken from GenBank.

The length of the β6 segment was found to be 2364 bp. Comparison with homologous proteins in the BLAST database showed that suckling mouse β6 had a high degree of homology with *Mus musculus* integrin β6 (99.0%) (Accession no.: NM_ 021359). The amino acid sequence of suckling mouse β6 also showed a high degree of homology with its human, pig, dromedary, Bactrian camel, sheep, rabbit, rat, horse, dog, rhesus macaque, bovine, hylobates leucogenys, white-tufted-ear marmoset and little brown bat counterparts (Figure 1): 90.0%, 88.0%, 88.0%, 89.0%, 88.0%, 89.0%, 96.0%, 90.0%, 90.0%, 90.0%, 89.0%, 90.0%, 90.0% and 89.0%, respectively.

Using EMBOSS/pepstates (v6.0.1), we predicted that the protein isoelectric point was 5.1415, the molar extinction coefficient reaches 55900. ScanProsite was used to predict the protein motif: two EGF-like extracellular domains (479–490 bp and 563–574 bp) and two cysteine-rich extracellular domains (511–524 bp and 591–604 bp) were found. CBS Prediction Servers/Signal 3.0 Server online showed that the protein contains a putative signal peptide comprising 21 residues (1–21). Using EMBOSS/tmap and TMHMM Server v. 2.0, the protein was predicted to include an extracellular domain of 687 residues (22–706 bp), a transmembrane domain of 25 residues (707–732 bp), and a cytoplasmic tail of 56 residues (733–788 bp). Using the NetNGlyc 1.0 Server and EMBOSS/antigenic (v6.0.1), we found that the extracellular domain also includes 9 (9/10) possible *N*-linked glycosylation sites and 29 (29/31) possible antigenic sites. 

**Figure 2:**
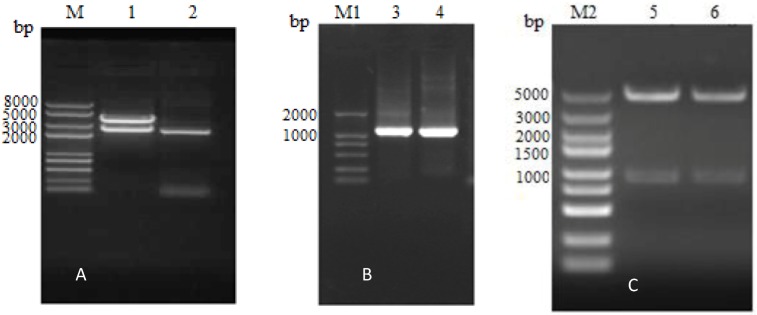
Identificationof recombinant plasmids. (A) Lane M represents the DNA marker DL8000 and gel electrophoresis results for the pGEX-β6 recombinant plasmid by enzyme digestion (lane1) and PCR (lane 2): the length of β6 was 2364 bp and the length of the pGEX-4T-1 vector segment was about 4900 bp. (B) Lane M1 represents the DNA marker DL2000 and gel electrophoresis results for pETβ6-1 (lane3) and pETβ6-2 (lane 4) amplified by PCR: the length of β6-1 was 1077 bp and the length of β6-2 was 1080 bp. (C) Lane M2 represents the DNA marker DL5000 and gel electrophoresis results for pETβ6-1 (lane5) and pETβ6-2 (lane 6) after enzyme digestion: the length of β6-1, β6-2 were 1077 and 1080bp, respectively; the length of pET30a vector segment was about 5400 bp.

The integrin β6 extracellular domain was subsequently amplified as two separate segments. The first segment (designated β6-1­) is 1077 nucleotides in length. The second segment (designated β6-2­) contains 1080 nucleotides, after the elimination of added ATG and TAA, and also encodes a 358-amino acid fragment. As shown in Figure 2B and C, the β6-1 and β6-2 segments were cloned into a pET30a vector to generate pETβ6-1 and pETβ6-2.

### 2.2. Expression and Purification of Integrin β6-1 and β6-2

**Figure 3:**
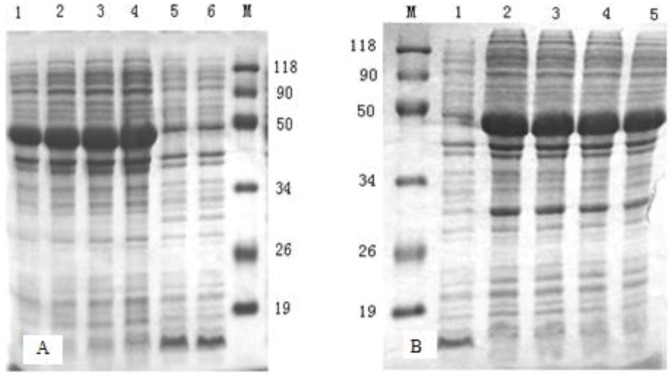
SDS-PAGE analysis. (A) SDS-PAGE analysis of β6-1 product. Lane M represents the low-molecular-weight marker. Lanes 1–4 represent the products atdifferent incubation times (4, 5, 6, and 7 h) after induction with pETβ6-1. Lane 5 represents the vector control culture. Lane 6 represents the non-induced control culture. (B) SDS-PAGE analysis of β6-2 product. Lane M represented the low-molecular-weight marker. Lane 7 represents the non-induced control culture. Lanes 8–11 represent the products at different incubation times (4, 5, 6, and 7 h) after induction with pETβ6-2.

**Figure 4:**
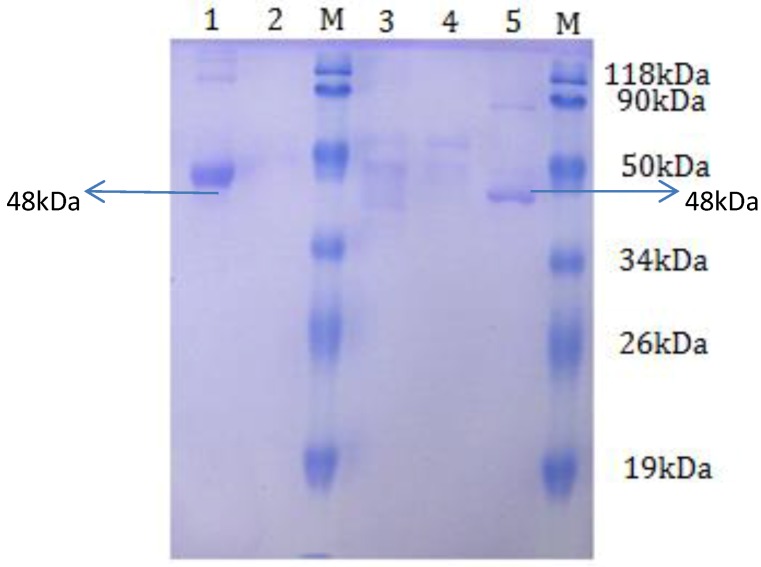
SDS-PAGE analysis of purified β6-1 and β6-2. Lane M represents the low-molecular-weight marker. Lane 1 represents the purified product of pETβ6-1 recombinant protein. Lanes 2 and 3 represent the purified product of the non-induced control culture. Lane 4 represents the purified product of the vector control culture. Lane 5 represents the purified product of pETβ6-2 recombinant protein.

*Escherichia coli* BL21cells were transformed with the recombinant plasmids pETβ6-1 and pETβ6-2. To obtain high expression of the proteins, different incubation times after induction were compared after transformation. As shown in Figure 3, the expression level of the proteins increased with an increase in incubation time. The level of expression was slightly higher at 6 h after induction for β6-1 and 5 h after induction for β6-2. After purification, SDS-PAGE revealed a single clear band for both fusion proteins, at about 48 kDa (Figure 4).

### 2.3. T Sell Response in Animals Vaccinated with Integrin β6 Extracellular Domains

**Figure 5:**
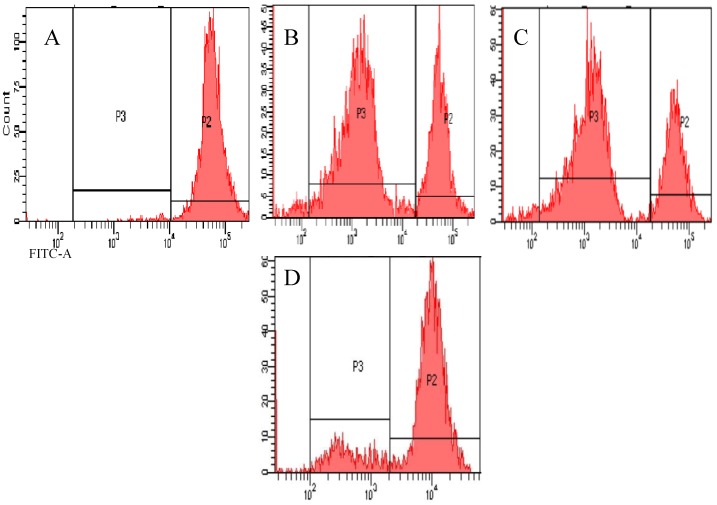
Proliferation of β6-1- and β6-2-stimulated lymphocytes. (A) Unlabeled and unstimulated cells were used as the negative control. (B) Proliferation of T lymphocytes by PHA stimulation (positive control). (C) Proliferation of T lymphocytes in response to β6-1 stimulation. (D) Proliferation of T lymphocytes in response to β6-2 stimulation.

Animals were vaccinated with phosphate-buffered saline (PBS) (Group A), O-type FMD vaccine (Group B), β6-1 protein segment (Group C), β6-2 protein segment (Group D), or a mixture of β6-1 and β6-2 (Group E). Peripheral blood samples were taken at 0, 1, 2, 3, 4 and 5 weeks after the first immunization to determine T cell proliferation. Carboxyfluorescein diacetate succinimidyl ester (CFSE) fluorescence results showed that stimulation with β6-1 and phytohemagglutinin (PHA) resulted in significant proliferation of T lymphocytes, but the proliferation was not significant with β6-2 (Figure 5). 

**Figure 6:**
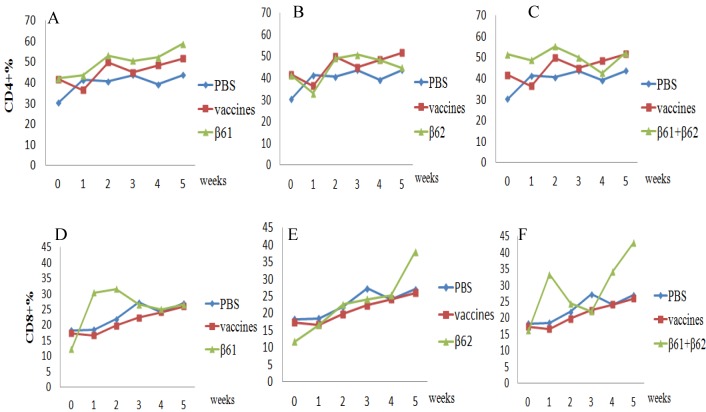
Cell-mediated immune response in guinea pigs vaccinated with PBS, conventional O-type foot-and-mouth disease (FMD) vaccine, β6-1, and β6-2. The upper panels show CD4^+^ lymphocyte proliferation in β6-1-inoculated guinea pigs (A), β6-2-inoculated guinea pigs (B), and β6-1+β6-2-inoculated guinea pigs (C) compared to the PBS- and vaccine-inoculated animals. The lower panels show CD8^+^ lymphocyte proliferation in β6-1-inoculated (D), β6-2-inoculated (E), and β6-1+β6-2-inoculated (F) guinea pigs compared to the PBS- and vaccine-inoculated animals.

The kinetics of CD4^+^ and CD8^+^ T lymphocytes were also analyzed by flow cytometry. As shown in Figure 6, the percentage of CD4^+^ cells in Group C was significantly greater than that in the PBS group from two weeks; it was also higher than the percentage in the vaccine group. In the groups given β6-2 and both protein segments, the percentage of CD4^+^ cells increased slightly compared to PBS group. In Group D, the percentage of CD8^+^ cells significantly increased at one week post vaccination (wpv), peaked at 2 wpv and then declined from 3 wpv. While the percentage of CD8^+^ cells of other two groups (Group E and Group F) had no obvious regularity.

### 2.4. In Vivo Effect of Induction of Anti-Integrin β6 Response on FMDV Infection

**Figure 7:**
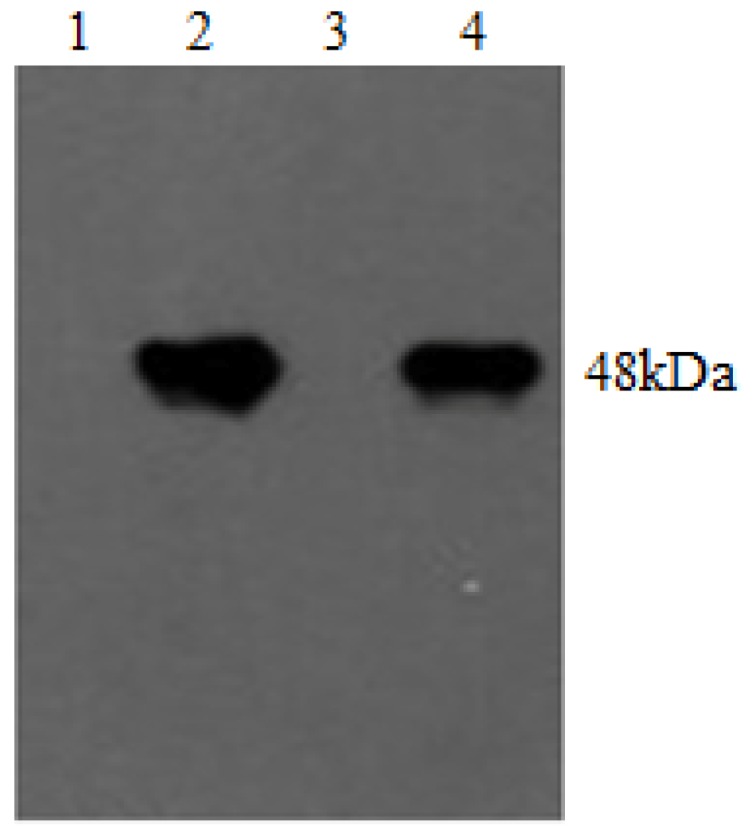
Western blot analysis of serum antibodies. Lanes 1 and 3 represent the negative control; lanes 2 and 4 represent inmmune signals for the recombinant protein pET-β6-1 and -β6-2 present in guinea pig anti-serum at two weeks after the second vaccination, respectively.

Serum antibodies against integrins were detected by western blot analysis. Immune response signals for the recombinant proteins pET-β6-1 and pET-β6-2 and guinea pig serum antibodies against β6-1 and β6-2 were both very strong (Figure 7).

**Figure 8:**
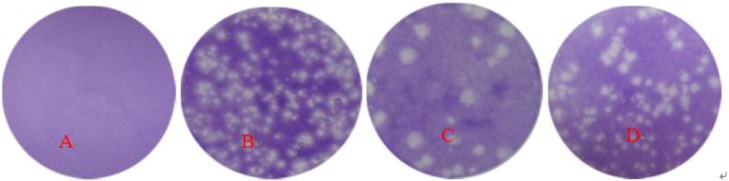
Plaque formation assay after FMD Virus (FMDV) challenge. (A) Negative control showing plaque formation in the absence of FMDV challenge. (B) Positive control showing plaque formation in response to FMDV challenge without anti-β6-1/β6-2 serum inoculation. (C) Plaque formation in the presence of anti-β6-1 protein serum. (D) Plaque formation in the presence of anti-β6-2 protein serum.

Serum antibodies against FMDV infection by the plaque formation assay. The results showed that when CHO-K1-αvβ6 cells were incubated with sera containing anti-β6-1 and anti-β6-2 antibodies and inoculated with FMDV, the anti-β6-1 serum could inhibit plaque formation apparently (Figure 8C), while the inhibitory effect of the anti-β6-2 serum was not very apparent (Figure 8D).

### 2.5. Results of Viral Challenge of the Inoculated Guinea Pigs

As summarized in Table 1, after the FMDV challenge, all the animals in Group A (control) developed severe lesions on both rear feet, while none of the guinea pigs in Group B (FMD vaccine) showed clinical signs. In Group D (β6-2), three of four guinea pigs had severe lesions on both rear feet. In Group E (β6-1 + β6-2), one of the four guinea pigs had no lesions and one had mild lesions on the rear feet, the other two had severe lesions on both rear feet. Interestingly, in Group C (β6-1), two of the four guinea pigs had no lesions and one had mild lesions on the rear feet, the fourth had severe lesions on both rear feet. Compared to the control group, the rate of protection offered by β6-1 was higher than that of β6-2 and β6-1+β6-2. 

Integrins contribute to a variety of biological processes, including cell proliferation, morphology, migration, and apoptosis [21]. In this study, we have investigated the role of integrin β6 extracellular domains β6-1 and β6-2 in the neutralization of FMDV infection in vitro in CHO-K1-αvβ6 cells and in vivo in guinea pigs.

We have described the cloning and sequencing of suckling mouse β6 and the subsequent expression of β6 extracellular domains, since it has been shown that αvβ6 interacts with viruses with high affinity. In addition, we divided the extracellular domain of the β6 subunit into two parts, β6-1­ (RGD-binding segment) and β6-2, and sequenced both segments. This is in keeping with the primary goal of many structure-function analyses in the integrin field, which is the reduction of macromolecular ligands to minimal recognition sequences [22]; many bioactive amino acid sequences have been teased out of large extracellular matrix proteins, especially those involved in FMDV binding and infection of β6-transfected cells, which is mediated through an RGD-dependent interaction [16]. Sequencing of mouse integrin-β6 subunit revealed close homology (~90%) with its human counterpart, which has previously been demonstrated to confer susceptibility to FMDV infection when expressed in the normally non-permissive cell line SW480 [16]. The sequence of suckling mouse integrin β6 shares all the main characteristics with the corresponding sequence of other animals. 

**Table 1:** Protection and symptom severity in guinea pigs given the viral challenge.

Serial number of guinea pigs	Group A (PBS)	Group B (Vaccine)	Group C (β6-1)	Group D (β6-2)	Group E (β6-1+β6-2)
Protection					
1	None	Total	Total	Partial	Total
2	None	Total	Total	None	Partial
3	None	Total	Partial	None	Partial
4	None	Total	None	None	Partial
Severity of symptoms					
1	Severe	None	None	Mild	None
2	Severe	None	None	Severe	Mild
3	Severe	None	Mild	Severe	Severe
4	Severe	None	Severe	Severe	Severe
Rate of protection (%)	0 (0/4)	100 (4/4)	50 (2/4)	0 (0/4)	25(1/4)

Both protein segments, β6-1 and β6-2, were expressed in a prokaryotic system (E. coli BL21). The expression of heterologous proteins in E. coli is widely employed for laboratory and preparative purposes with their lower cost and convenience [23,24]. Many proteins can now be produced routinely in secreted form with yields in the gram/liter scale [25]. Some of the most impressive examples of high expression yields for secreted proteins include the production of insulin-like growth factor I (IGF-I) as periplasmic inclusion bodies at 8.5 g/L (a value that represents the highest E. coli secreted protein yield reported in the literature) [26]. We chose this system over a eukaryotic one only because prokaryotic systems involve lower costs, high expression yields and are fast and easy to operate.

Guinea pigs were inoculated with both protein segments, and we found, from the results of the plaque formation assay, that the serum of animals inoculated with the β6-1 protein segment could remarkably neutralize FMDV. Thus, its use in FMD vaccines needs to be explored. Screening and pre-testing of many FMDV vaccines are conducted in guinea pigs because the cost is cheaper and the experimental results are similar to cattle; moreover, guinea pigs are easily susceptible to the FMDV. Therefore, we chose guinea pigs to evaluate proliferation of T lymphocytes and interference of the β6 subunits with FMDV infection. CD4^+^T cell responses are suggested to play an important role in protection against FMDV [27]. In this study as well, we found that the level of CD4^+^T cells in guinea pigs immunized with the β6-1 protein was higher than that in guinea pigs immunized with β6-2; this means that the ligand-binding domain of integrin subunits might play an important role in cell proliferation.

When β6-1 or β6-2 protein from suckling mice was used to inoculate mice, many mice experienced adverse symptoms such as skin ulcers and mental depression, and many even died (data not published); however, no apparent symptoms were noticed when guinea pigs were inoculated with these proteins. We think that the reaction in the mice may have been an autoimmune one. The host and source animal should not be of the same species, therefore, to prevent autoimmune reactions to cause damages to animals themselves, when testing integrin subunit proteins as anti-FMDV drugs, we choose suckling mice integrin subunit, not from bovine or pig source. 

To the best of our knowledge, FMDV evolution is strongly influenced by high mutation rates and shows quasispecies dynamics [28]. Recently, sub-neutralizing levels of soluble secreted αvβ6 were used as selective pressure to select resistant FMDV mutants: A-type isolates showed mutations in the normally conserved RGD motif or just outside of it within the G-H loop of VP1, while O-type isolates showed mutations in VP3 and residues proximal to the VP1 RGD motif [29]. In this study, integrin subunits acted as a blockade and reduced formation of αvβ6 heterodimers on the cell surface; thus, there was no opportunity for the formation of soluble integrin-resistant mutants, and there was no suitable binding region on the cell for the virus. Therefore, there was no chance of infection with resistant viruses. This is in agreement with the results of this study, which show that partial protection against FMDV infection was induced in the guinea pigs vaccinated with the integrin β6 extracellular domain containing the RGD-binding site (β6-1­). Although inactivated FMD vaccines have been available for decades, there is little or no cross-protection across serotypes and subtypes, which means that vaccines need to be matched specifically to circulating field strains [30]. A previous study has indicated that integrin blockade has the potential of blocking the αvβ6 receptor to which the FMDV binds. Thus, an integrin blockade may be able to interfere with FMDV infection in vivo. This raises the possibility that targeting integrin in vivo may enable the generation of a new strategy to control FMDV infection. 

## 3. Experimental Section

### 3.1. Animals

We used suckling mice as the source for integrin because they are inexpensive and easily available. Four healthy suckling mice (2–3 days of age) and thirty-six guinea pigs (adult, female, weighing 600–800 g) were obtained from Lanzhou Veterinary Research Institute. The guinea pigs were fed in an isolated hutch. They were maintained on a 12-h light and 12-h dark cycle with the temperature controlled at 23℃ with a relative humidity between 50 and 55%. All animal experiments were performed according to protocols approved by the animal care committee of our institute.

### 3.2. Virus

FMDV (O/MYA98/BY/2010) particles were serially diluted seven times (10-fold each time). To measure 50% infection dose (ID_50_) value for each virus, 0.2 ml of each of the seven dilutions was intramuscularly injected into two guinea pigs. Control animals were inoculated with 0.2 ml DMEM medium. 

### 3.3. Generation of Plasmids Encoding Soluble Mmouse Integrin β6 Subunits

Tongue and lung tissues were collected from suckling mice immediately after execution by cervical dislocation. Tissues were ground thoroughly with an RNase-free, liquid-nitrogen-cooled mortar and pestle. Total RNA from each tissue was extracted with an RNeasy Mini Kit (Qiagen, Germany) according to the manufacturer’s instructions. 

PCR primers for amplification of integrin β6 (Table 1) were designed based on nucleotide similarities between the available GenBank sequences for *Mus musculus*. RT-PCR was performed according to the manufacturer’s instructions (PrimeScript One Step RT-PCR Kit Ver.2, TaKaRa), with an annealing temperature of 58°C. The RT-PCR product was analyzed using a Bio-Rad gel imaging system and puriﬁed using the Qiaquick Gel Extraction Kit (Qiagen, Germany) according to the manufacturer’s instructions. The purified product was ligated into a pGEX-4T-1 vector (it was preserved in our laboratory), and the resultant recombinant plasmids were confirmed to contain the β6 subunits by PCR, restriction enzyme digestion and sequencing. Sequence analyses were performed using the BLAST search program of the National Center for Biotechnology Information. The sequence identity analyses were carried out using the Laser-gene analysis software package (DNASTAR, USA). Phylogenetic trees were constructed based on the nucleotide sequences using the neighbor-joining algorithm of MEGA version 5.05 (Arizona State University) [31]. The reference sequences included in the analysis were taken from GenBank: *Mus musculus* (NM_ 021359), pig (A4GUC1), dromedary (C9E0L7), Bactrian camel (A5Z1X7), sheep (B0FYY3), rabbit (G1T891), rat (Q6AYF4), horse (F7B3K7), dog (E2RBL9), *Rhesus macaque* (F7HQ11), human (P18564), bovine (Q8SQB8), *Hylobates leucogenys* (G1QM06), white-tufted-ear marmoset (F6PKM6), little brown bat (G1PC35).

The correct recombinant vector was designated as pGEX-β6. Subsequently, the segments β6-1 and β6-2 of the integrin β6 extracellular domains were separately amplified from pGEX-β6 by PCR using the specific primers (Table 1). The reactions were as follows: disintegration at 94°C for 2 min; this was followed by 35 cycles at 94°C for 30 s, and 30 s of annealing at the respective temperatures for the integrin segments to be ampliﬁed (Table 1) and 2 min at 72°C; this was subsequently followed by a final extension phase for 10 min at 72°C. The amplified products were verified by 1% agarose gel electrophoresis and analyzed using a gel imaging system (PC Module, BIO-RAD). The amplification products of the segments β6-1 and β6-2 were puriﬁed and then ligated into the pET30a vector (it was preserved in our laboratory). The resultant recombinant plasmids were confirmed to be correct by restriction enzyme digestion (*BamHI* and *XholI*, Table 1) and sequencing. The correct recombinant expression plasmids were designated as pETβ6-1 and pETβ6-2 and used for protein expression.

The structure of the integrin subunits was characterized using EMBOSS/pepstates (v6.0.1), EMBOSS/antigenic (v6.0.1), EMBOSS/tmap (European Molecular Biology Open Software Suite，EMBOSS). ScanProsite, CBS Prediction Servers/Signal 3.0 Server online, and TMHMM Server v. 2.0, and the NetNGlyc 1.0 Server, following the link: http://www.cbs.dtu.dk/services/.

**Table 2:** Primers used in the cloning of the integrin β6 subunit.

Primers	Sequence(5′to 3′)	Target gene	Predicted size	Annealing temperature
β6F	5′- *CGGGATCC* ATGGGGATTGAGCTGGTCTGCCTGT-3′	Integrin β6 subunit	2364 bp	58.0°C
β6R	5′- *CCGCTCGAG*CTACCCATCTGAAGAAAGGCCCACTT-3′			
β6-1F	5′- *CGGGATCC*ATGGGGATTGAGCTGGT-3′	5′ region of integrin β6 extracellular domains	1077 bp	54.5°C
β6-1R	5′- *CCGCTCGAG*TTACCCAGAATCCTTCTGAA-3′			
β6-2F	5′- *CGGGATCC*ATGACCGTGGGACTGCTTCAGA-3′	3′ region of integrin β6 extracellular domains	1080 bp	57.0°C
β6-2R	5′- *CCGCTCGAG*TTAGTTTGGAGGTTTGGGGCAGT-3′			

“_”: enzyme digestion site. β6-1 and β6-2 overlap by 30 bp

### 3.4. Expression and Purification of Integrin β6-1 and β6-2

*E coli* BL21 cells were transformed with the recombinant plasmid pETβ6-1 or pETβ6-2. Transformed cells were incubated in lysogeny broth (LB) at 37°C till they reached the mid-log phase, and they were then induced with 5 mM Isopropyl β-D-1-Thiogalactopyranoside（IPTG）(Quantum Scientific, Murarrie, QLD, Australia) for different incubation times (4, 5, 6 and 7 h) at 28°C. Bacterial cultures were harvested by centrifugation at 5000 × *g* for 10 min at 4°C. The expression yield was measured using standard SDS-PAGE. The non-induced control culture and the pET30a vector control culture were analyzed in parallel.

Recombinant integrin extracellular domains β6-1 and β6-2 were purified from bacterial cultures with high expression yield. Briefly, cells were lysed by incubation with 0.05% lysozyme (Sigma–Aldrich) at 4°C and then sonicated after the addition of the protease inhibitor cocktail (Sigma–Aldrich) and Phenylmethanesulfonyl fluoride (PMSF) (Sigma–Aldrich). Inclusion bodies were solubilized with 4 M urea for 4 h at 4°C, and then centrifuged at 6000 × *g* for 10 min at 4°C. The supernatant was harvested and purified using the BugBuster His·Bind purification kit (Merck, German) as described by the manufacturer. Purified recombinant integrin β6-1 and β6-2 segments were quantified using the BCA protein assay (Thermo Scientific, Scoresby, VIC, Australia).

### 3.5. Immunization with the β6-1 and β6-2 Subunits

The guinea pigs were divided into five groups at random, each containing four guinea pigs: Group A, 0.4 ml PBS with the conventional adjuvant (206) was given two times with a two-week interval (negative control); Group B, 0.4 ml of conventional O-type FMD vaccine with the same adjuvant was given once; Group C, 0.4 mg of the β6-1 protein was given with the adjuvant two times with a two-week interval; Group D, 0.4 mg of the β6-2 protein was given as described for Group C; Group E, 0.4 mg of β6-1 and β6-2 mixture, which both proteins in equal amounts, was given as described for Group C. Guinea pigs were inoculated intramuscularly at the tibialis cranialis in both rear legs. 

### 3.6. T-lymphocyte Proliferation Assay

Whole blood was collected by heart puncture at 0 (1day before immunization), 1, 2, 3, 4 and 5 weeks after the first immunization, and 1 ml of the sample was diluted with 1ml PBS. The following procedures were performed in the dark. Sufficient carboxyfluorescein diacetate succinimidyl ester (CFSE) stock solution (Sigma) was added directly to make a final concentration of 10µg/ml. The suspension was vortexed immediately following CFSE addition to ensure rapid dispersal, and then left to incubate for 30 min at 37°C. The dyeing reaction was terminated by the addition of cold RPMI 1640 complete medium containing 10% fetal calf serum (Gibco). Labeled cells were transferred to 12-well plates, with 1 ml of cell suspension in each well. In order to induce cell division, they were cultured at three days. The divided cells were then incubated with PHA (10 µg/ml, Sigma) and β6-1 or β6-2 protein (10 µg/ml) for another three days. Unlabeled and unstimulated cells were used as negative controls. To assess proliferation, the cells were cultured in a CO_2_ incubator (37°C, 5% CO_2_, dark) for 3 days, after which they were harvested and transferred to a flow tube (12 × 75 mm polystyrene tubes). Then, 2 ml of erythrocyte lysis buffer was added, and the solution was centrifuged at 450 *g* for 10 min at room temperature. The cell precipitate was washed twice with 4 ml PBS (0.01 M, pH 7.4). Finally, the cells were re-suspended in 400μl PBS (0.01 M, pH 7.4). FACS Aria Flow cytometry (BD, USA) was used to detect the proliferation results, and FACSDiva software (BD, USA) used to obtain the data. 

### 3.7. Detection of CD4^+^ and CD8^+^ T-lymphocytes

To detect CD4^+^ and CD8^+^ T-lymphocytes, 100μl whole blood was directly stained as follows: FITC-labeled anti-mouse CD8 antibody and phycoerythrin (PE)-labeled anti-mouse CD4 antibody (BD Pharmingen) were incubated with the cells for 20 min at room temperature in the dark. Then, an appropriate volume of erythrocyte lysing solution (Beijing Solarbio Science & Technology Co. Ltd., China) was added to the tubes, and after vortexing thoroughly, the cells were incubated for 15 min on ice and centrifuged at 450 × *g* for 10 min at 4°C. The supernatant was aspirated and the cells were washed twice with PBS (0.01 M, pH 7.4). Finally, the cells were re-suspended in 400 μl PBS (0.01 M, pH 7.4) and analyzed by flow cytometry. The samples were stored at 4°C in the dark until flow cytometric analysis. Data analysis was carried out using the BD FACSAria software.

### 3.8. Detection of Serum Antibodies

Serum was isolated from the whole blood collected from guinea pigs vaccinated with the β6-1 and β6-2 proteins at two weeks after the second vaccination. Serum antibodies against integrins were detected by western blot analysis. After SDS-PAGE, the gel strips were transferred to nitrocellulose membranes. The membranes were blocked with 5% fetal bovine serum (FBS) overnight at 4°C. They were then incubated with a 1:1000 dilution of the serum for 1 h at RT. Rabbit anti-guinea pig IgG peroxidase conjugate (Sigma) at a 1:3000 dilution was then added for 1 h at RT. Then, the WestDura SuperSignal chemiluminescent reagent (Pierce) was added and the strips were visualized on an X-ray film (X-Omat; Kodak, N.Y., USA).

### 3.9. Effect of Neutralizing Antibodies on Induction of FDMV Infection *In vivo*

To determine whether induction of neutralizing antibodies can inhibit proliferation of FMDV in vitro, we performed a plaque formation inhibitory assay using the serum of guinea pigs vaccinated with the β6-1 and β6-2 proteins at two weeks after the second vaccination. CHO-K1 cell lines which only expressed αvβ6 (preserved in our laboratory, designated as CHO-K1-αvβ6) were seeded at 1–2 × 10^6^ cells per well in six-well plates and incubated for 16 h at 37°C with 5% CO_2_. Cell monolayers were washed with PBS (pH 7.5) containing 2mM CaCl_2_ and 1mM MgCl_2_. Then, the cells were divided into four groups. Two groups of cells were incubated with PBS (200 µl/well); one group was incubated with a 100-fold dilution of the anti-β6-1 protein serum (200 µl/well); and the fourth group was incubated with a 100-fold dilution of the anti-β6-2 protein serum (200 µl/well). Except for the first group, cells of the other groups were inoculated with 200 µl FMDV (10-fold serial dilution in PBS containing 1% fetal calf serum). Plaque formation by FMDV in CHO-K1-αvβ6 cells was characterized by the standard plaque assay [32,33]. Infectious centers were visualized as plaques by ﬁxation and crystal violet staining.

### 3.10. FMDV Challenge in Immunized Guinea Pigs

All guinea pigs were challenged with 0.2 ml of 100 ID_50_ of live virus injected subcutaneously into the back of the sole at the third week after the second vaccination. All guinea pigs were kept in isolated hutches and examined daily for seven days. Severity of lesions on both rear feet was recorded daily. The disease severity was scored as follows: no infection, no lesions on both rear feet; mild infection, lesions on one rear foot; severe infection, lesions on both rear feet. Based on the clinical scores recorded above, protection was scored as described in [34]: total protection, complete absence of lesions; partial protection, lesions restricted to one rear foot; no protection, lesions on both rear feet. The rate of protection (%) was calculated as the percentage of the number of guinea pigs with no lesions on both rear feet divided by the total number of guinea pigs examined.

### 3.11. Statistical Analysis

The data were analyzed with SPSS 13.0 (Statistical Product and Service Solutions, Chicago). P values less than 0.05 were considered to indicate statistical significance. 

## 4. Conclusions

We found evidence for the role of integrin extracellular subunit β6-1 from suckling mice in neutralizing FMDV infection in guinea pigs. We think that this lays the foundation for future strategies against FMDV infection. 
